# A homogenized constrained mixture model of cardiac growth and remodeling: analyzing mechanobiological stability and reversal

**DOI:** 10.1007/s10237-023-01747-w

**Published:** 2023-07-23

**Authors:** Amadeus M. Gebauer, Martin R. Pfaller, Fabian A. Braeu, Christian J. Cyron, Wolfgang A. Wall

**Affiliations:** 1 Institute for Computational Mechanics, Technical University of Munich, 85748 Garching, Germany; 2 Pediatric Cardiology, Stanford Maternal & Child Health Research Institute, and Institute for Computational and Mathematical Engineering, Stanford University, Stanford, USA; 3 Ophthalmic Engineering & Innovation Laboratory, Singapore Eye Research Institute, Singapore National Eye Centre, Singapore, Singapore; 4 Singapore-MIT Alliance for Research and Technology, Singapore, Singapore; 5 Yong Loo Lin School of Medicine, National University of Singapore, Singapore, Singapore; 6 Institute of Continuum and Material Mechanics, Hamburg University of Technology, 21073 Hamburg, Germany; 7 Institute of Material Systems Modeling, Helmholtz-Zentrum Hereon, 21502 Geesthacht, Germany

**Keywords:** Cardiac growth and remodeling, Homogenized constrained mixture model, Computational modeling, Mechanobiology, Hypertension

## Abstract

Cardiac growth and remodeling (G&R) patterns change ventricular size, shape, and function both globally and locally. Biomechanical, neurohormonal, and genetic stimuli drive these patterns through changes in myocyte dimension and fibrosis. We propose a novel microstructure-motivated model that predicts organ-scale G&R in the heart based on the homogenized constrained mixture theory. Previous models, based on the kinematic growth theory, reproduced consequences of G&R in bulk myocardial tissue by prescribing the direction and extent of growth but neglected underlying cellular mechanisms. In our model, the direction and extent of G&R emerge naturally from intra- and extracellular turnover processes in myocardial tissue constituents and their preferred homeostatic stretch state. We additionally propose a method to obtain a mechanobiologically equilibrated reference configuration. We test our model on an idealized 3D left ventricular geometry and demonstrate that our model aims to maintain tensional homeostasis in hypertension conditions. In a stability map, we identify regions of stable and unstable G&R from an identical parameter set with varying systolic pressures and growth factors. Furthermore, we show the extent of G&R reversal after returning the systolic pressure to baseline following stage 1 and 2 hypertension. A realistic model of organ-scale cardiac G&R has the potential to identify patients at risk of heart failure, enable personalized cardiac therapies, and facilitate the optimal design of medical devices.

**Supplementary Information:**

The online version contains supplementary material available at 10.1007/s10237-023-01747-w.

## Introduction

Cardiac growth and remodeling (G&R) occur in various situations throughout a human’s life. Developmental growth occurs between birth and adulthood and ensures the adaption of the cardiac function to the changing needs. Changing needs also occur in adulthood, for example, due to pregnancy or exercise resulting in physiological growth of the myocardium. Disease-induced stimuli, however, often result in pathologic, maladaptive G&R that develop toward heart failure. Common diseases stimulating pathologic G&R are myocardial infarction, aortic stenosis, hypertension, or valvular regurgitation (Cohn et al. 2000). This paper will focus on pathological G&R in the adult heart.

### Phenotypes of cardiac G&R in heart failure

Two measures are commonly used to characterize different cardiac G&R patterns: The mass of the left ventricle (LV) and the ratio of LV wall thickness to the diastolic diameter, denoted as relative wall thickness. With these two measures, cardiac G&R can be classified into two distinct patterns:

If both the relative wall thickness and LV mass are increased, the pattern is classified as *concentric hypertrophy*. This pattern is typically observed in patients with increased resistance to ejection, e. g., aortic stenosis or hypertension. On the cellular level, contractile-protein units (sarcomeres) in cardiomyocytes are added in parallel so that the length to width ratio of individual cardiomyocytes decreases (Kehat and Molkentin 2010).

An increase in LV mass with constant relative wall thickness is classified as *eccentric hypertrophy*. This pattern is typically a result of valvular insufficiencies (Linzbach 1960). Cardiomyocytes increase their length to width ratio by serial addition of sarcomeres (Kehat and Molkentin 2010).

Ventricular and cellular G&R in the heart also involves changes in the collagen network that surround each myocyte (Kehat and Molkentin 2010). Increased collagen fraction in the myocardium, i. e., myocardial fibrosis, results in a higher stiffness and causes poor diastolic filling characteristics (Spinale 2007), increasing the risk of heart failure (Brower et al. 2006). Additionally, fibrosis impairs contractility and electrophysiology, causing arrhythmias, local microfibrillations, and inefficient contraction (Schirone et al. 2017). Increased collagen deposition is mainly observed in pressure overloaded cases. In contrast, a loss of collagen fibrils is observed in cases with volume overload (Spinale 2007), supporting the dilatory process of the ventricle (Kehat and Molkentin 2010).

### Fundamentals of G&R

We will only briefly discuss the mechanobiological processes behind G&R of soft tissue. The interested reader is referred to the review of Cyron and Humphrey (2017) and references therein.

#### Homeostasis

It is generally accepted that there is a preferred mechanical state within living tissue that mechanobiological activity seeks to retain (Eichinger et al. 2021b). This state is often referred to as homeostatic state.

In vitro studies with collagen-gels seeded with fibroblasts support this hypothesis. The initially stress-free gels develop internal stresses that reach a plateau after a few hours. A subsequent stretching or relaxing of the gels, i.e., a perturbation from the apparently homeostatic state, immediately changes the stress level followed by a slow, exponential return back toward the homeostatic plateau (Eichinger et al. 2021a; Cyron and Humphrey 2017; Brown et al. 1998; Ezra et al. 2010).

Cardiomyocytes are known to react to increased loading conditions by synthesizing new contractile proteins and assembling new sarcomeres (Cohn et al. 2000) to adapt their size. Yang et al. (2016) have shown in in vitro experiments that new sarcomeres were assembled within the cell. Although in vitro studies of homeostasis of cardiomyocytes are rare, it is plausible that also cardiomyocytes have some preferred mechanical state that they try to maintain. Given their high volume fraction and their importance during systole, Grossman et al. (1975) support this with their broadly accepted wall stress hypothesis that states that myocardial wall thickness increases to return systolic stress to normal.

#### Turnover

Turnover is a key underlying process by which G&R occurs in living soft tissue. It is the continuous deposition and active degradation of tissue constituents (Humphrey and Rajagopal 2002). In the physiological case, it maintains tissue integrity and prevents mechanical fatigue. The amount of deposited (secreted) mass is balanced with the actively degraded mass so that overall tissue mass and structure do not change (tissue maintenance). In case of injury or disease, there can be imbalance resulting in effective increase or decrease of tissue mass to return constituent stresses back to homeostasis.

Turnover rates of cardiomyocytes are low: Fewer than $50\,\%$ of cardiomyocytes are replaced during lifetime in a healthy heart (Bergmann et al. 2009). Less than $1\,\%$ of cardiomyocytes in a healthy adult heart are replaced per year (Bergmann et al. 2015). However, to maintain cardiac function, there exists an orchestrated intracellular process that synthesizes, assembles and degrades proteins of the sarcomeres inside cardiomyocytes (Willis et al. 2009). Turnover in the context of cardiomyocytes can therefore be seen as an intracellular process (Humphrey and Rajagopal 2002).

The collagen network that surrounds cardiomyocytes also turnover. Around $0.6\,\%$ of the collagen is synthesized per day in the healthy state (Weber 1989) by fibroblasts (Humphrey 2002). Their half-life time is estimated to be 80 to $120\,{\textrm{days}}$ (Weber 1989). In case of injury, a six- to eightfold increase in collagen synthesis is reported (Weber 1989).

### Mathematical models of cardiac G&R

Many different mathematical models have been proposed so far in the field of cardiac G&R. We only discuss a few of them. The interested reader is instead referred to the review papers of Lee et al. (2016), Aboelkassem et al. (2019), Yoshida and Holmes (2020), Niestrawska et al. (2020), and Sharifi et al. (2021).

An important group of models originates from the kinematic growth theory introduced by Rodriguez et al. (1994). They propose a multiplicative split of the deformation gradient into an inelastic, growth-related part and an elastic part. Kroon et al. (2009) used this model to simulate inhomogeneous 3D isotropic volumetric growth of a truncated ellipsoid. Since then, the model has been modified and extended several times. For example, Göktepe et al. (2010) simulated concentric and eccentric growth, Lee et al. (2015) extended the model to capture also reversal of growth. This model has also been used to reconstruct growth features from in vivo MRI (Fan et al. 2021) and has been coupled with 0D models of stimuli from hormonal signals (Estrada et al. 2020).

As Humphrey and Rajagopal (2002) pointed out, kinematic growth theory mainly models certain consequences of growth. It captures the outcome of G&R only phenomenologically (Niestrawska et al. 2020) instead of modeling the fundamental ongoing processes in living tissue. For example, such models limit growth by artificially introducing a maximal pathological myocyte inelastic stretch or limiting the number of growth steps. In a predictive model of cardiac G&R, however, mechanobiological stability (Cyron and Humphrey 2014; Cyron et al. 2014) of the G&R process must be a result of the geometry, tissue properties, and severity of the pathological event, rather than of a priori assumptions. As Yoshida and Holmes (2020) point out, simple kinematic growth models also often fail to predict reverse growth that is expected after the removal of the pathological load (e. g., repair of an aortic stenosis).

A fundamentally different approach has been proposed by Humphrey and Rajagopal (2002). They model soft tissue as a mixture of different constituents. Mass increments of constituents are deposited at every point in time into the mixture and existing mass is degraded over time — as observed in the mechanobiology of tissue turnover. Hence, G&R is a consequence of modeling fundamental processes observed in living tissue. The model, so far, has mostly been applied to vascular G&R. Due to the computational costs of the model evaluation and the complexity of the model, 3D models of G&R are rare. Cyron et al. (2016) proposed a temporal homogenization of all mass depositions for each constituent resulting in a drastic reduction of the computational costs. The so-called homogenized constrained mixture model has been applied to 3D vascular G&R on a thick-walled cylinder (Braeu et al. 2016) and patient-specific geometries (Mousavi et al. 2019). To the best of our knowledge, constrained mixture models have not been applied to cardiac G&R yet.

### Outline

In this paper, we propose a homogenized constrained mixture model for cardiac G&R that bases on the principal mechanobiological processes described above. We demonstrate the capabilities of the model on a truncated ellipsoid model of an idealized LV to capture mechanobiological stability and reversal of cardiac G&R. Finally, we discuss the results and outline possible further steps for improvement.

## Mathematical modeling

Let $\mathscr {B}_0$ be the reference configuration of a body at time^1^$s_0=0$ and $\mathscr {B}_s$ the configuration at time *s*. A material point $\varvec{X} \in \mathscr {B}_0$ is mapped to its current position $\varvec{x} \in \mathscr {B}_s$ via

$$\begin{aligned} \varvec{x}: \mathscr {B}_0 \times [0, \infty ) \rightarrow \mathscr {B}_s, \quad (\varvec{X}, s) \mapsto \varvec{x}(\varvec{X}, s). \end{aligned}$$

The displacement field is $\varvec{u}=\varvec{x}-\varvec{X}$ and the deformation gradient is $\varvec{F} = \frac{\partial \varvec{x}}{\partial \varvec{X}}$ with the Jacobian determinant $J = \det \varvec{F}$.

G&R occurs on very long time scales so that inertial effects can be neglected. Using the principle of virtual work (Holzapfel 2000), the static mechanical equilibrium can be written as

1 $$\begin{aligned} \delta W = \int _{\mathscr {B}_0} \varvec{P} : \delta \varvec{F} \, \textrm{d}V = 0, \end{aligned}$$

where $\varvec{P}$ is the first Piola–Kirchhoff stress tensor.

### Microstructural modeling of the myocardium

We model the myocardium as a constrained mixture that consists of multiple structurally relevant constituents. All constituents *i* occupy all points $\varvec{x} \in \mathscr {B}_s$ and deform together with no relative motion between them ($\varvec{F} = \varvec{F}^i$). Quantities related to a specific constituent are denoted by superscript *i*.

G&R is modeled by a homogenized constrained mixture model as proposed by Cyron et al. (2016) and Braeu et al. (2016, 2019). In contrast to classical constrained mixture models (Humphrey and Rajagopal 2002), the deposition and degradation of mass increments are captured only in a temporally homogenized sense. Instead of capturing the natural configuration of every mass increment, only an averaged inelastic deformation of each constituent $\varvec{F}_\text {r}^i$ is stored. The total deformation can be written as

2 $$\begin{aligned} \varvec{F} = \varvec{F}_\text {e}^i \varvec{F}_\text {r}^i, \end{aligned}$$

where $\varvec{F}_\text {e}^i$ is the elastic deformation of constituent *i* that ensures that (1) is satisfied and all constituents deform together.

The first Piola–Kirchhoff stress tensor $\varvec{P}$ in (1) is

3 $$\begin{aligned} \varvec{P} = \frac{\partial \varPsi }{\partial \varvec{F}}, \end{aligned}$$

with the strain energy (per unit volume)

4 $$\begin{aligned} \varPsi = \sum _{i=0}^n \rho _0 ^i W^i \left( \varvec{C}_\text {e}^i\right) + \varPsi ^\#. \end{aligned}$$

$W^i$ is the strain energy per unit mass, $\rho _0^i$ is the reference mass density of constituent *i*, and $\varvec{C}_\text {e}^i = \varvec{F}_\text {e}^{i\text {T}} \varvec{F}_\text {e}^i$ is the Cauchy–Green deformation tensor of the elastic deformation of constituent *i*. This homogenization of the stress response across all constituents is denoted as *rule-of-mixtures* (Humphrey and Rajagopal 2002). Only the equilibrium for the whole mixture has to be solved and not for each constituent individually. $\varPsi ^\#$ is a penalty-type strain energy function per unit volume to ensure a (nearly) constant spatial density of the whole tissue at any time (incompressibility).

The strain energy functions of the constituents are only functions of the constituent’s elastic deformation. Deformation that stems from the inelastic deformation (i.e., G&R) does not store elastic energy and therefore does not directly create stresses.

We assume that the primarily structurally relevant constituents of the myocardium are collagen fibers ($i=\text {c}$) and cardiomyocytes ($i=\text {m}$). Both constituents are modeled to be quasi-1D constituents that only bear stresses in their preferred direction. The strain energy per unit mass of the collagen fibers is an exponential of the form

5 $$\begin{aligned} W^\text {c} = \frac{a^\text {c}}{2b^\text {c}} \left\{ \exp \left[ b^\text {c} \left( I_{4}^\text {c}-1\right) ^2\right] -1 \right\} , \end{aligned}$$

where $I_{4}^\text {c} = \varvec{f}_r^\text {c} \cdot \varvec{C}_\text {e}^\text {c} \varvec{f}_r^\text {c}$ is the fourth pseudo-invariant of $\varvec{C}_\text {e}^\text {c}$ (Holzapfel 2000) and $\varvec{f}_r^\text {c}$ is the preferred direction of the collagen fiber family in the intermediate configuration (related to the reference configuration via $\varvec{F}_\text {r}^\text {c}$).

For cardiomyocytes, we use an additively decomposed strain energy function

6 $$\begin{aligned} W^\text {m} = W_\text {pas}^\text {m} + W_\text {act}^\text {m}, \end{aligned}$$

to incorporate passive and active contributions, respectively. The passive contribution is modeled with an exponential of the form

7 $$\begin{aligned} W_\text {pas}^\text {m} = \frac{a^\text {m}}{2b^\text {m}} \left\{ \exp \left[ b^\text {m} \left( I_{4}^\text {m}-1\right) ^2\right] -1 \right\} , \end{aligned}$$

analogously to collagen fibers.

Active muscle tone has not been developed yet for cardiomyocytes in the G&R timescale. We, therefore, adopt a simple model from Wilson et al. (2012) for vascular smooth muscle tone via an additional term in the strain energy function:

8 $$\begin{aligned} W_\text {act}^\text {m} = \frac{\sigma _{\text {act}}^\text {m}}{\rho _0(s=0)} \left( \lambda _\text {act}^\text {m} + \frac{1}{3} \frac{(\lambda _\text {max}^\text {m}-\lambda _\text {act}^\text {m})^3}{(\lambda _\text {max}^\text {m}-\lambda _0^\text {m})^2} \right) . \end{aligned}$$

$\sigma _{\text {act}}^\text {m}$ is the maximal active Cauchy stress, $\lambda _\text {act}^\text {m}$ is the active stretch in fiber direction, and $\lambda _0^\text {m}$ and $\lambda _\text {max}^\text {m}$ are the active stretch at zero and maximum active stress. In accordance with Wilson et al. (2012), we assume that the active muscle tone evolves with the G&R of cardiomyocytes, such that $\frac{\partial \lambda _{\text {act}}^\text {m}}{\partial \lambda ^{\text {m}}} = \frac{1}{\lambda ^\text {m}}$, where $\lambda ^\text {m}$ is the current total stretch of the muscle fiber compared to the reference configuration (Braeu et al. 2019).

Collagen fibers and cardiomyocytes are excluded during compression^2^ ($I_4^i<1$) since their contribution to compressive stresses is usually assumed to be negligible (Holzapfel and Ogden 2009).

The remaining structural constituents (mainly elastin; $i=\text {3D}$) are modeled with a decoupled isotropic neo-Hookean strain energy contribution:

9 $$\begin{aligned} W^\text {3D} = c_1 \left( \bar{I}_1-3\right) . \end{aligned}$$

$\bar{I}_1 = J^{-{2}/{3}} \text {tr}{\left( \varvec{G}^{\text {3D}} \varvec{F}^\text {T}\varvec{F} \varvec{G}^{\text {3D}}\right) }$ is the first modified invariant (Holzapfel 2000) of the total elastic Cauchy–Green deformation tensor of elastin including the isochoric and rotation-free prestretch tensor of elastin $\varvec{G}^\text {3D}$. The prestretch tensor maps the stress-free configuration of elastin into the reference configuration.

In the following, we will introduce the needed evolution equations for G&R. Our model currently does not consider direct interactions between constituents; hence, we can formulate these evolution equations for each constituent separately.

### Mass production and removal

As described earlier, the continuous deposition and degradation of mass are called tissue turnover. Turnover is modeled as a superposition of deposition and degradation of mass. We therefore can split the net mass production rate $\dot{\rho }_0^i$ into a true mass production rate $\dot{\rho }_{0+}^i$ and a true mass degradation rate $\dot{\rho }_{0-}^i$:

$$\begin{aligned} \dot{\rho }_0^i = \dot{\rho }_{0+}^i + \dot{\rho }_{0-}^i. \end{aligned}$$

In homeostasis, the deposition exactly compensates for the degradation of mass. We track the deposition and degradation of mass by an evolution of the mass density in the *reference* configuration. Hence, the amount of mass in a reference volume element changes over time, and mass is conserved between the evolved reference configuration and the current configuration.

We assume the following constitutive relation for the net mass production rate:

10 $$\begin{aligned} \dot{\rho }_{0}^i = \rho _{0}^i k^i \frac{\sigma ^i-\sigma _\text {h}^i}{\sigma _\text {h}^i}, \end{aligned}$$

where $\sigma ^i$ is the current Cauchy stress of constituent *i* in their preferred direction, $\sigma _\text {h}^i$ is the preferred homeostatic stress state and $k^i$ is a growth gain factor.

The degradation of mass is assumed to follow a simple Poisson process

11 $$\begin{aligned} \dot{\rho }_{0-}^i (s) = -\frac{\rho _0^i(s)}{T^i}, \end{aligned}$$

where $T^i$ is the mean lifetime of a deposited mass increment.

### Remodeling

**Fig. 1:**
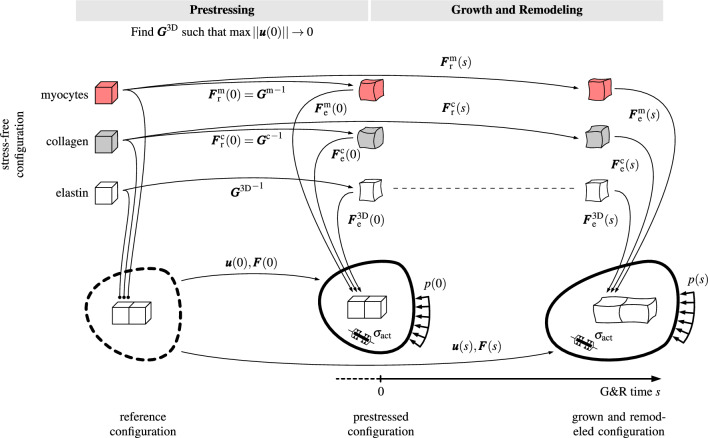
The different configurations during prestressing, and growth and remodeling. Each configuration consists of multiple constituents, namely cardiomyocytes, collagen fibers and elastin, which occupy every point in the domain at once. The reference configuration is obtained from imaging data of a heart in systole. The initial condition for the remodeling deformation gradients of cardiomyocytes and collagen fibers is chosen such that these constituents are in their preferred mechanical environment in the reference ($\widehat{=}$ imaged) configuration. Prestress, active stress and external loading cause the mixture to elastically deform. The prestress algorithm aims at an elastin prestretch tensor $\varvec{G}^\text {3D}$ such that the prestressed and the reference configurations are identical ($\varvec{u}(0)=\varvec{0}$), or at least similar. The prestressed configuration, therefore, is in (or close to) mechanobiological equilibrium. A subsequent pathological event (e.g., an increase of the pressure *p*) triggers growth and remodeling of the tissue. The stress-free configurations of cardiomyocytes and collagen fibers evolve through the continuous deposition and degradation of mass increments that are captured in a homogenized sense in $\varvec{F}_\text {r}^{i}(s)$

Remodeling of the tissue is a direct consequence of continual turnover. At every point in time, new mass increments of cardiomyocytes and collagen fibers are deposited and extant constituents continuously degrade. New mass increments of constituent *i* deposit at rate $\dot{\rho }_{0+}^i$ and are assembled in general in a different stress state than the extant material of the constituent. Extant mass is continuously degraded at a rate of $\dot{\rho }_{0-}^i$. This deposition and degradation of mass locally change the stress state of the constituent, resulting in a rearrangement of the constituent which is remodeling of the tissue.

Homogenized constrained mixture models capture the mass increments and their stress-free configurations in a temporally homogenized sense by an adaption of the stress-free configuration of the constituent (see Fig. 1). We assume herein that new mass increments are deposited and assembled in their preferred mechanical state $\varvec{\sigma }_{\text {h}}^i$ and extant mass of the constituent is continuously degraded with the current Cauchy stress $\varvec{\sigma }^i$. Cyron et al. (2016) and Braeu et al. (2016) have shown that this change in the stress-free configuration can be described by an evolution of an inelastic part of the deformation gradient with the equation

12 $$\begin{aligned} \left[ \frac{\partial \varvec{\sigma }^i}{\partial \varvec{F}_\text {e}^i} : \left( \varvec{F}_\text {e}^i \varvec{L}_\text {r}^i\right) \right] _{\varvec{F}=\text {const.}} = \left( \frac{\dot{\rho }_0^i (s)}{\rho _0^i} + \frac{1}{T^i} \right) \left( \varvec{\sigma }^i-\varvec{\sigma }_{\text {h}}^i\right) , \end{aligned}$$

where $\varvec{L}_\text {r}^i = \dot{\varvec{F}}_\text {r}^i {(\varvec{F}_\text {r}^i)}^{-1}$ is the remodeling velocity gradient. The stiffness $\frac{\partial \varvec{\sigma }^i}{\partial \varvec{F}_\text {e}^i}$ for the quasi-one-dimensional fiber families as defined in Eq. (7) is rank deficient. Hence, the remodeling deformation modes that do not contribute to strain energy can be chosen arbitrarily. If we assume incompressible remodeling and that newly deposited fibers are always aligned in the same direction in the reference configuration, the rotational part of $\varvec{F}_\text {r}^i$ is the identity matrix. We can then write (Cyron et al. 2016)

13 $$\begin{aligned} \varvec{F}_\text {r}^i = \lambda _\text {r}^i \,\varvec{f}_0^i \otimes \varvec{f}_0^i + \frac{1}{\sqrt{\lambda _\text {r}^i}} (\varvec{I}-\varvec{f}_0^i \otimes \varvec{f}_0^i), \end{aligned}$$

where the inelastic remodeling stretch in fiber direction $\lambda _\text {r}^i$ is the only unknown. A consequence of the rotation-free $\varvec{F}_\text {r}^i$ is that we can use $\varvec{f}_0^i$ which is the unit vector field in the *reference* configuration of the preferred direction of the fiber constituent. Equation (12) can then be simplified so that the only unknown $\lambda _\text {r}^i$ can be computed with (see Appendix 1 in Cyron et al. (2016) for details of the derivation)

14 $$\begin{aligned} \dot{\lambda }_\text {r}^i = \left[ \frac{\dot{\rho }_0^i}{\rho _0^i} + \frac{1}{T^i}\right] \frac{\lambda _r^i}{2\ I_4^i} \left[ \frac{\partial \sigma ^i}{\partial I_4^i}\right] ^{-1}\left( \sigma ^i - \sigma _\text {h}^i\right) . \end{aligned}$$

Equation (14) can be integrated in time at every Gauss point using a standard time integration scheme starting from an initial condition. As simulations are often started from a homeostatic state, the initial remodeling stretch is chosen such that the elastic stretch of the constituent initially equals the homeostatic stretch. Using Eq. (2) and $\varvec{F}(s=0)=\varvec{I}$ (identity matrix), we get

$$\begin{aligned} \lambda _\text {r}(s=0) = \frac{1}{\lambda _\text {h}^i}. \end{aligned}$$

**Fig. 2:**
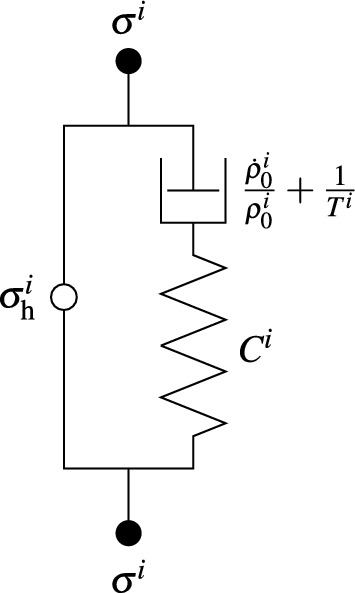
The physical analog model of a homogenized constrained mixture model subjected to turnover as shown by Cyron et al. (2016). A Maxwell element (right) with a spring with stiffness $C^i$ in and dashpot with time constant $\frac{\dot{\rho }_0^i}{\rho _0^i}+\frac{1}{T^i}$ parallel to a motor unit (left) exerting the homeostatic (pre-)stress $\sigma _\text {h}^i$

Cyron et al. (2016) derived a physical interpretation of turnover in homogenized constrained mixture models. They showed that a Maxwell element with a parallel motor unit as shown in Fig. 2 is a mechanical analog model. The Maxwell element is a spring with some nonlinear stiffness and a dashpot with time constant $\frac{\dot{\rho }_0^i}{\rho _0^i}+\frac{1}{T^i}$ in series. The motor element exerts the homeostatic stress $\sigma _\text {h}^i$. When the current stress state deviates from the homeostatic stress state of the constituent, the deviation has to be borne by the Maxwell element. Maxwell elements are inherently unable to support static loads resulting in an isochoric shift of the stress-free length of the constituent.

In the kinematic growth theory, G&R is captured by defining an inelastic G&R tensor phenomenologically. Here, the remodeling deformation gradient is derived from the ideas of the full constrained mixture by temporal homogenization of mass turnover. This model is mechanobiologically motivated with computational costs comparable to the kinematic growth theory.

### Growth

By now, we have modeled continual turnover of the constituents and how it causes an inelastic (isochoric) remodeling. Still missing is the modeling of the volume change due to mass increase or decrease, that is, growth.

There are different approaches that describe the volume change due to mass growth. An often chosen approach is to define an additional inelastic deformation $\varvec{F}_\text {g}^i$ for each constituent that incorporates the volume change of the tissue. The definition of such an inelastic deformation typically requires the definition of a structural tensor that defines the anisotropy of growth. As obtaining this information from imaging data is difficult, anisotropy of growth is often chosen such that the obtained results are reasonable. This hinders the approach from being truly predictive.

Braeu et al. (2019) proposed a way that bypasses the controversial definition of the growth tensor. They assumed that the deposition of new mass results in an elastic distention of whole differential volume elements also affecting other constituents. Due to the high stiffness of collagen fibers and cardiomyocytes, this elastic distention mainly occurs perpendicular to the existing fibers. Remodeling according to (12) transforms this (in general) anisotropic distension over time into an inelastic deformation.

We are following this approach and modify the penalty type strain energy term $\varPsi ^\#$ that approximates incompressibility so that it nearly ensures a constant spatial density:

15 $$\begin{aligned} \varPsi ^\# = \frac{\kappa }{2} \left( |\varvec{F}|-\frac{\rho _0(s)}{\rho _{0}(s=0)}\right) ^2. \end{aligned}$$

The current reference mass density $\rho _0$ of the whole tissue is

$$\begin{aligned} \rho _0(s) = \sum _{i=0}^n \rho _0^i(s). \end{aligned}$$

When choosing the penalty parameter $\kappa$ sufficiently high, a nearly constant spatial density is ensured. The minimization of the total strain energy will then result in a dilation mainly in the direction with the smallest stiffness. The interested reader is referred to Braeu et al. (2019), who theoretically analyzed the natural anisotropic growth stemming from anisotropic stiffness of the tissue.

### Prestress

The geometry for a G&R simulation is usually a configuration obtained from magnetic resonance imaging (MRI) through segmentation. External loads present during imaging, e. g., from the blood pressure *p* and the surrounding tissue, result in an in vivo reference configuration that is not stress-free. Since we model the tissue as a constrained mixture, each constituent can have its own (incompatible) stress-free configuration. Note that it is necessary that the in vivo configuration is not stress-free such that all constituents can be in their homeostatic state for a given left ventricular pressure *p*.

In our examples, we assume that the imaged configuration represents a healthy state before some physiological event occurs that stimulates G&R. The configuration must therefore be in mechanobiological equilibrium, which requires mechanical equilibrium and additionally that all constituents are in their preferred homeostatic mechanical state (Cyron and Humphrey 2014). As cardiomyocytes occupy the largest volume fraction in cardiac tissue, we motivate the selection of the reference configuration based on them. We heuristically assume that cardiomyocytes attain their homeostatic state during systole, where their generated active force is at maximum. Note that tensional homeostasis of tissue under pronounced cyclical loading remains poorly understood to date and the definition of such a configuration is part of ongoing research.

The constituents subject to G&R are the cardiomyocytes and the collagen fibers. Both are quasi-1D fiber constituents. They are assumed to be in their homeostatic state if their current stress state equals their preferred stress state ($\sigma ^i = \sigma _\text {h}^i$) that is known and a-priori defined. We will define this preferred state using the homeostatic stretch $\lambda _\text {h}^i$ obtaining the homeostatic stress from the constitutive relation.

Cocciolone et al. (2018) found that synthesis of functional elastin in vessels mainly happens during development and maturation. Lacking of specific experiments, we assume similar behavior in the myocardium. Our assumption is that elastin does not have a mechanism to maintain its preferred stress state in maturity. Therefore, even in mechanobiological equilibrium, the stress response of elastin is non-homogeneous in contrast to the stress responses of cardiomyocytes and collagen fibers. Figure 1 depicts the different configurations during prestressing. The prestress algorithm aims for the elastin prestretch tensor $\varvec{G}^\text {3D}$ such that the prestressed configuration is identical (or at least similar) to the reference ($\widehat{=}$ imaged) configuration. The prestretch tensor of collagen fibers and cardiomyocytes is given by

$$\begin{aligned} \varvec{G}^i = \lambda _\text {h}^i \varvec{f}_0^i \otimes \varvec{f}_0^i + \frac{1}{\sqrt{\lambda _\text {h}^i}} \left( \varvec{I} - \varvec{f}_0^i \otimes \varvec{f}_0^i \right) , \end{aligned}$$

such that these constituents are in their preferred mechanical state in the reference configuration

Mousavi and Avril (2017) and Weisbecker et al. (2014) introduced an iterative algorithm that we will adopt with a modification: Since only the deviatoric stretches contribute to the internal energy of elastin, we choose $\varvec{G}^\text {3D}$ to be isochoric and rotation-free (symmetric).

To ease the solution of the nonlinear equilibrium equations, the external forces and the a-priori defined prestretch of the fibrillar constituents are applied gradually in the first couple of timesteps. Our initial guess is that elastin is stress-free in the reference configuration, hence $\varvec{G}_{0}^\text {3D} = \varvec{I}$. After every prestress iteration *k*, we compute the new isochoric prestretch deformation gradient

$$\begin{aligned} \bar{\varvec{F}}_{k+1,\text {pre}} = \bar{\varvec{F}} \varvec{G}_k^\text {3D}, \end{aligned}$$

with $\bar{\varvec{F}} = \left( J\right) ^{-\frac{1}{3}} \varvec{F}$ being the isochoric part of the deformation gradient. To ensure that the prestretch tensor is rotation-free, a polar decomposition is computed yielding the new prestretch tensor $\varvec{G}_{k+1}^\text {3D}$:

$$\begin{aligned} \varvec{G}_{k+1}^\text {3D} = \varvec{R}_{k+1,\text {pre}}^\text {T}\bar{\varvec{F}}_{k+1,\text {pre}}, \end{aligned}$$

where $\varvec{R}_{k+1,\text {pre}}$ is the rotational part of $\bar{\varvec{F}}_{k+1,\text {pre}}$.

This iterative algorithm is repeated until the maximum Euclidean norm of the nodal displacements between the reference and the prestressed configuration falls below a tolerance $\varepsilon _\text {pre}$. This tolerance ensures that the maximum deviation of the prestressed configuration from the reference ($\widehat{=}$ imaged) configuration is bounded. It can be chosen depending on the resolution of the imaging data and the acceptable difference between these configurations. It is important to note that the displacements after prestressing also influence the mechanobiological equilibrium of the 1D constituents. A subsequent phase of G&R can achieve mechanobiological equilibrium at the cost of additional displacements and, therefore, a further deviation from the reference configuration.

### Solving the G&R problem

The resulting model of G&R is a quasi-static problem given by Eq. (1) with local evolution equations given by Eq. (10) and (14) on each integration point. The proposed model can be easily implemented in many existing computational frameworks of classical solid mechanics as a constitutive model. To compute the stress given the deformation gradient of the mixture at each integration point, the following steps have to be done:

1. Integrate the local evolution Eqs. (10) and (14) using a standard time integration scheme.
2. Compute the remodeling deformation gradient of each constituent using Eq. (13).
3. Compute the elastic part of the deformation of each constituent by Eq. (2).
4. Compute the stress response of the mixture with Eqs. (3)–(9) and (15).

The nonlinear equilibrium Eq. (1) is solved with a Newton–Raphson type algorithm. Within that, the steps above have to be done in every iteration. If an explicit time integration is used in step 1, steps 1 and 2 have to be done only once per timestep.

## Numerical examples

**Fig. 3:**
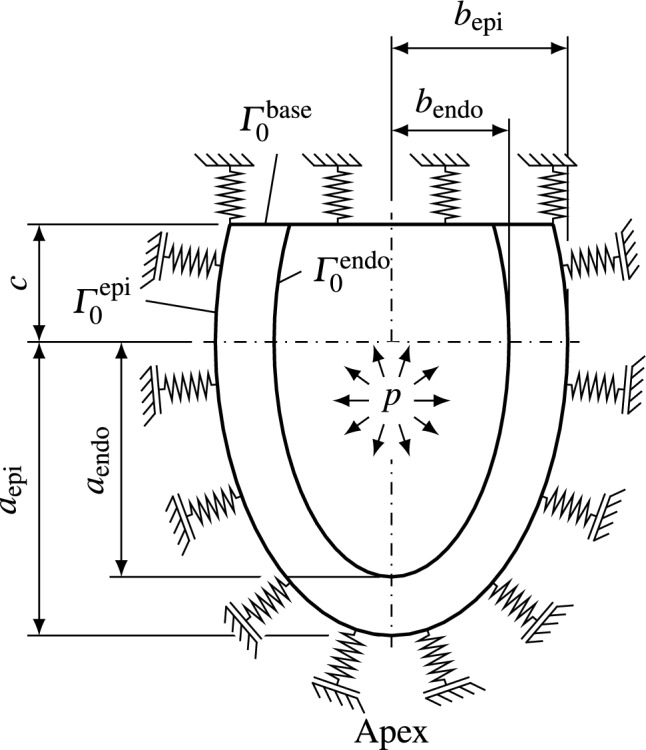
The prolate spheroid used as a model left ventricle for the simulation of G&R of the myocardium. The base of the heart is fixed with omnidirectional springs and the pericardium is modeled with springs in reference normal direction to mimic the stiffness of the left atrium and the surrounding tissue. The ventricle is loaded with systolic pressure *p*

We implemented the above model in the in-house research code BACI (2021) written in C++. As often done in the literature, we used the prolate spheroid shown in Fig. 3 as a simple model of a left ventricle. We assume that cardiomyocytes and collagen fibers seek homeostasis in the systolic configuration. The dimensions of the ventricle, therefore, represent a human LV in systole. We performed a mesh refinement study and identified a mesh with 3970 quadratic tetrahedral elements to yield a good tradeoff between computational efficiency and accuracy. The mesh was generated with Gmsh (Geuzaine and Remacle 2009). We use a Newton algorithm with backtracking to solve nonlinear systems of equations and a conjugate gradient method for linear systems of equations (The Trilinos Project Team 2021).

**Fig. 4:**
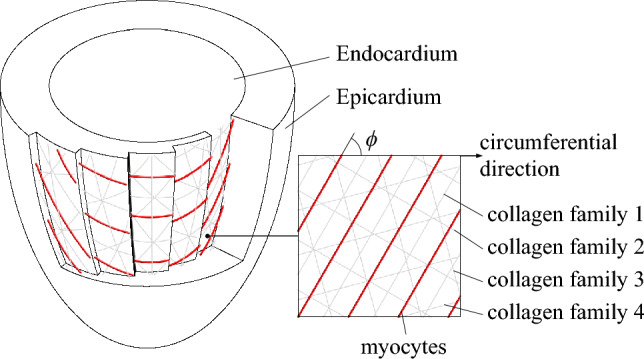
The fiber distributions within the prolate spheroid. The helix angle $\phi$ of Cardiomyocytes (red) varies from $+60^\circ$ at the endocardium to $-60^\circ$ at the epicardium. The four discrete collagen fiber families are aligned in myocyte fiber, cross-fiber and diagonal ($\pm 45^\circ$) directions

The helix angle $\phi$ of cardiomyocytes varies continuously from $+60^\circ$ at the endocardium to $-60^\circ$ at the epicardium. We used the method described by Nagler et al. (2017) to obtain the fiber directions of cardiomyocytes at every point in the domain. Collagen fibers are represented with four discrete fiber orientations that are aligned in the wall plane in myocyte fiber, cross-fiber, and diagonal ($\pm 45^\circ$) direction (see Fig. 4). The mass fractions for each collagen fiber direction are equally distributed.

The evolution Eqs. (10) and (14) are integrated with a forward Euler scheme using a step size that is a twentieth of the minimum mean survival time ($T^i$ in Eq. (11)). We verified the integration of the local evolution equations with an implicit trapezoidal rule to exclude any numerical artifacts. All obtained results are identical within a small tolerance.

Figure 3 visualizes the geometry and the boundary conditions. To mimic the support of the left atrium, the base of the heart is supported by omnidirectional springs. The epicardium is supported with springs in reference surface normal direction to capture the influence of the pericardium and surrounding tissue (Pfaller et al. 2019). These springs are also prestressed during the prestress algorithm as outlined in Pfaller et al. (2019). On the endocardium, we apply a pressure to represent the systolic pressure of the blood. The resulting virtual work formulation is

16 $$\begin{aligned} \begin{aligned} \delta W =&\int _{\mathscr {B}_0} \varvec{P} : \delta \varvec{F}\, \textrm{d}V\\ +&\int _{\varGamma _0^\text {base}} c_\text {base}\varvec{u} \cdot \delta \varvec{u} \, \textrm{d}\varGamma + \int _{\varGamma _0^\text {epi}} (c_\text {p} \varvec{u} \cdot \varvec{N}_0) \varvec{N_0} \cdot \delta \varvec{u} \, \textrm{d}\varGamma \\ +&\int _{\varGamma _0^\text {endo}} p J \varvec{F}^{-\text {T}} \varvec{N}_0 \cdot \delta \varvec{u} \, \textrm{d}\varGamma = 0, \end{aligned} \end{aligned}$$

where $\varvec{N}_0$ is the reference outward surface normal, $\varGamma _0^\text {base}$ is the cut-off surface of the ventricles, $\varGamma _0^\text {epi}$ and $\varGamma _0^\text {endo}$ are the epicardial and endocardial surfaces, respectively.

The parameters used for the simulation can be found in Table 1. Material parameters for constrained mixture type models of the myocardium have not yet been developed. Hence, we adopted the material parameters and homeostatic stretches from Braeu et al. (2016) who developed a model of vascular G&R. The mean survival time of a sarcomere is estimated from the half-life time of the respective proteins (Willis et al. 2009). The stiffness of the pericardial boundary condition is taken from Pfaller et al. (2019). The initial mass fractions are roughly estimated from reported volume fractions in Holzapfel and Ogden (2009). The dimensions of the used geometry were obtained from an MRI image of a 33-year-old healthy female volunteer.

**Table 1:** Simulation parameters used to study a hypertensive heart and reverse remodeling

Name	Parameter	Value
*Geometry*		
Major epicardial radius	$a_\text {epi}$	$55\,{\textrm{mm}}$
Minor epicardial radius	$b_\text {epi}$	$30\,{\textrm{mm}}$
Major endocardial radius	$a_\text {endo}$	$46\,{\textrm{mm}}$
Minor endocardial radius	$b_\text {endo}$	$19\,{\textrm{mm}}$
Truncation	*c*	$13\,{\textrm{mm}}$
Epicardial fiber helix angle	$\phi_\text{epi}$	$-60^\circ$
Endocardial fiber helix angle	$\phi_\text{endo}$	$60^\circ$
*Material parameters*		
Myocytes: Fung exponential parameters	$a^\text {m}$	$7.6\,{{\textrm{J}}/{\textrm{kg}}}$
	$b^\text {m}$	11.4
Myocytes: Active muscle tone	$\sigma _{\text {act}}^\text {m}$	$54\,{\textrm{kPa}}$
	$\lambda _{0}^\text {m}$	0.8
	$\lambda _{\text {max}}^\text {m}$	1.4
Collagen: Fung exponential parameters	$a^\text {c}$	$568\,{{\textrm{J}}/{\textrm{kg}}}$
	$b^\text {c}$	11.2
Elastin: Neo-Hookean parameter	$a^\text {e}$	$72\,{{\textrm{J}}/{\textrm{kg}}}$
Volumetric penalty	$\kappa$	$150\,{\textrm{kPa}}$
*Homeostatic stretches*		
Myocytes: Homeostatic stretch	$\lambda _\text {h}^\text {m}$	1.1
Collagen: Homeostatic stretch	$\lambda _\text {h}^\text {c}$	1.062
*Boundary conditions*		
Base spring stiffness	$c_\text {base}$	$2.0\,{{\textrm{kPa}}/{\textrm{mm}}}$
Pericardial spring stiffness	$c_\text {p}$	$0.2\,{{\textrm{kPa}}/{\textrm{mm}}}$
Baseline systolic blood pressure	*p*	$120\,{\textrm{mmHg}}$
Stage 1 systolic blood pressure	*p*	$140\,{\textrm{mmHg}}$
Stage 2 systolic blood pressure	*p*	$180\,{\textrm{mmHg}}$
*Initial conditions*		
Total initial reference mass density	$\rho _0(s=0)$	$1050\,{{\textrm{kg}}/{\textrm{m}^3}}$
Initial myocyte mass fraction	$\xi ^\text {m}(s=0)$	0.6
Initial collagen mass fraction	$\xi ^\text {c}(s=0)$	0.1
Initial elastin mass fraction	$\xi ^\text {e}(s=0)$	0.3
Initial inelastic remodeling stretch	$\lambda _\text {r}^i(s=0)$	$\frac{1}{\lambda _\text {h}^i}$
*G&R parameters*		
Myocyte growth gain	$k^\text {m}$	${0.1}/{T^\text {m}}$
Myocyte sarcomere mean survival time	$T^\text {m}$	$10\,{\textrm{days}}$
Collagen growth gain	$k^\text {c}$	${0.1}/{T^\text {c}}$
Collagen mean survival time	$T^\text {c}$	$15\,{\textrm{days}}$
*Stability criteria*		
Homeostasis threshold	$\epsilon _{\sigma ,\text {h}}$	$10^{-3}$

The material parameters and homeostatic stretches are adopted from Braeu et al. (2016) who modeled vascular G&R. The mean survival time of sarcomeres is estimated from Willis et al. (2009). The stiffness of the pericardial boundary conditions is taken from Pfaller et al. (2019) and initial mass fractions are estimated from volume fractions in healthy myocardium (Holzapfel and Ogden 2009). The classification of hypertension is taken from Chobanian et al. (2003)

### Prestress

**Fig. 5:**
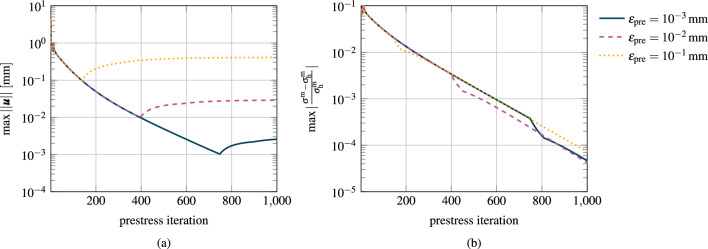
Convergence of the prestress algorithm. After prestretching, G&R is activated with unchanged boundary conditions. **a** Maximum Euclidean norm of the nodal displacements between the reference configuration and the prestressed configuration for different convergence thresholds. After the subsequent G&R phase, the maximum Euclidean norm of the nodal displacements is around half of an order of magnitude higher than the chosen prestress convergence thresholds. **b** Maximum relative deviation of the myocyte fiber Cauchy stress from the homeostatic stress for different convergence thresholds

**Fig. 6:**
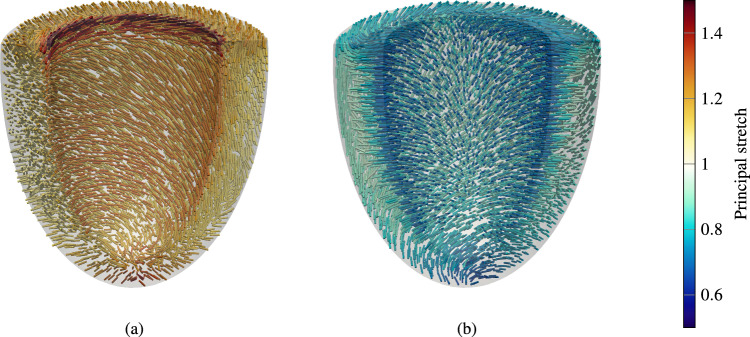
Visualization of the elastin prestretch. **a** Principal direction and principal stretch of the largest Elastin expansion (first principal stretch) and **b** for the largest compression (third principal stretch) at every integration point, respectively

In the following, we analyze the prestress algorithm presented in Sect. 2.5. The goal is to obtain the prestretch tensor of elastin at each Gauss point such that the reference configuration is in equilibrium with the systolic pressure $p=120\,{\textrm{mmHg}}$ applied at the endocardium. We linearly ramp up the pressure and the homeostatic stretch of the G&R constituents in the first 10 timesteps. We then run our prestressing algorithm until the maximum Euclidean norm of the nodal displacements falls below $\varepsilon _\text {pre}$. Finally, we terminate the prestressing algorithm and activate our G&R model to equilibrate any residual deviations from the homeostatic state.

We investigate 3 different prestress convergence thresholds $\varepsilon _\text {pre} \in \left\{ 10^{-1}, 10^{-2}, 10^{-3}\right\} \,{\textrm{mm}}$. Since the displacements of the prestressed configuration are not exactly zero, the constituents are not in perfect but approximate homeostasis. We thus also investigate in this section how the myocardium approaches a stable homeostatic configuration after convergence of the prestress algorithm.

Figure 5(a) shows the maximum Euclidean norm of the nodal displacements over the prestress iterations for each simulation and (b) shows the respective relative deviation from homeostasis for the cardiomyocytes.

After 136 timesteps, the maximum nodal displacement falls below the first threshold of $\varepsilon _\text {pre} = 0.1\,{\textrm{mm}}$. The remaining displacements cause a maximum relative deviation from homeostasis of the cardiomyocytes of $2\,\%$. When activating G&R at this stage, G&R converges toward a limit configuration with a final maximum nodal displacement of $0.4\,{\textrm{mm}}$.

If we choose the threshold $\varepsilon _\text {pre} = 0.01\,{\textrm{mm}}$ for the prestress algorithm, 393 iterations are needed to fulfill the criterion. The deviation from homeostasis for cardiomyocytes is $0.4\,{\%}$. The maximum nodal displacement after converged G&R is $0.03\,{\textrm{mm}}$.

The lowest investigated threshold $\varepsilon _\text {pre} = 0.001\,{\textrm{mm}}$ for the prestress algorithm is achieved after 745 iterations with $0.04\,{\%}$ deviation from homeostasis. The maximum nodal displacement after subsequent G&R is $0.003\,{\textrm{mm}}$.

Figure 5b depicts the maximum deviation from homeostasis in the myocardium during prestressing and the subsequent G&R phase. The convergence behavior toward mechanobiological equilibrium, characterized by the slope of the three curves, is similar in all three cases during prestressing and the subsequent G&R phase.

In the following, we analyze the prestretch of elastin. We analyze the case with $\varepsilon _\text {pre}=0.01\,{\textrm{mm}}$ after convergence of the prestress algorithm. Figure 6(a) depicts the direction of the largest principal elastin prestretch (largest elastin expansion) and (b) for the smallest principal stretch (largest elastin compression) at every integration point in the myocardium, respectively.

The largest principal stretch occurs at the basal plane at the endocardium, with a principal stretch of around 1.5. A slightly lower principal stretch occurs in the apical region. Generally, the principal stretches are larger at the endocardium and smaller at the epicardium. At the endocardium, elastin is under expansion in the plane of cardiomyocytes and collagen fibers, and under compression perpendicular to the plane (to bear the load of the blood pressure). On the epicardium, elastin is under expansion perpendicular to the fiber plane, where the springs of the pericardial boundary condition pull on the contracted ventricle (see Fig. 6).

### Mechanobiological stability

**Fig. 7:**
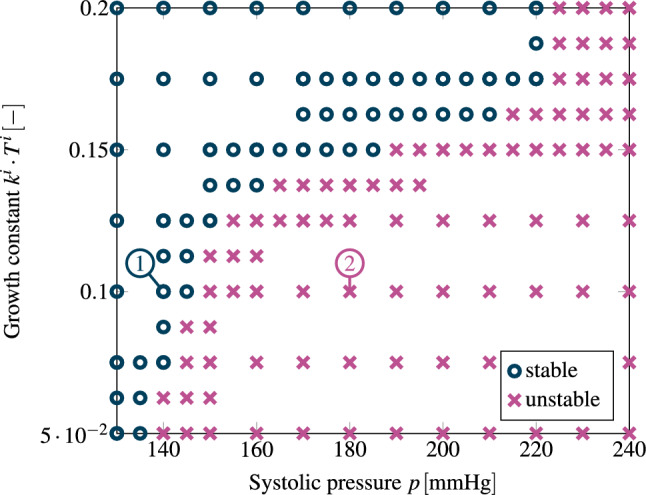
Mechanobiological stability map of heart growth depending on the systolic pressure and the growth constant $k^\text {i}\cdot T^i$. Blue circles mark simulations that result in a stable end configuration, whereas simulations with purple crosses mark cases with unbounded growth. The cases (1) and (2) are shown in more detail in Fig. 8

**Fig. 8:**
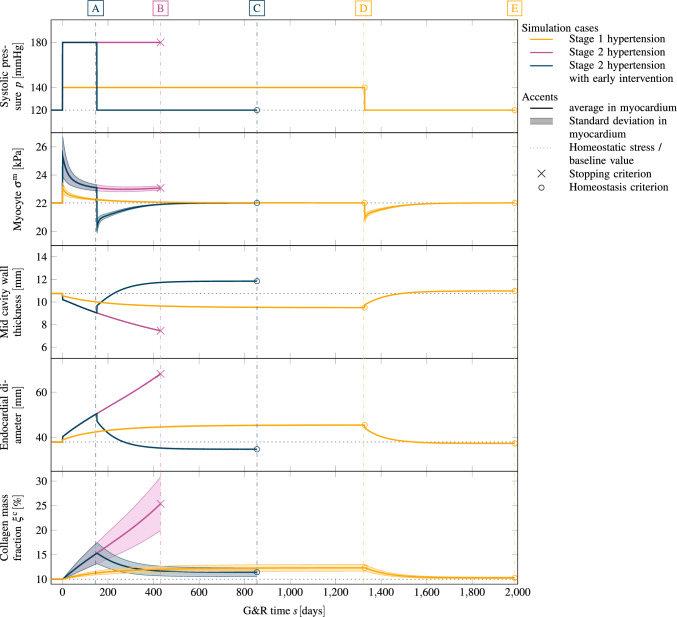
Stage 1 and stage 2 hypertension of a left ventricle with a subsequent pressure normalization and resulting reversal of G&R. For each case, we plot (from top to bottom) the applied systolic pressure, the resulting Cauchy stress of cardiomyocytes with standard deviation, the mid cavity wall thickness, the endocardial diameter and the collagen mass fraction with the standard deviation in the myocardium. Stage 1 hypertension resulted in stable G&R. After a normalization of the systolic pressure, all quantities return back close to their pre-G&R reference values. Both configurations **D** and **E** are depicted in Fig. 9. Stage 2 hypertension results in unstable G&R. After the deviation from homeostasis of all constituents consistently increased, the simulation is stopped. This configuration **B** is depicted in Fig. 10. If hypertension is treated after $150\,{\textrm{days}}$ (configuration **A**) the reverse G&R phase results in a stable configuration **C**. Mid cavity wall thickness, endocardial diameter and collagen mass fraction differ from the pre-G&R reference values. Both configurations **A** and **C** are depicted in Fig. 11. In the supplementary material, we also show the evolution of the Cauchy fiber stress of each collagen fiber family and the evolution of the mass fraction of cardiomyocytes and all collagen fiber families individually

**Fig. 9:**
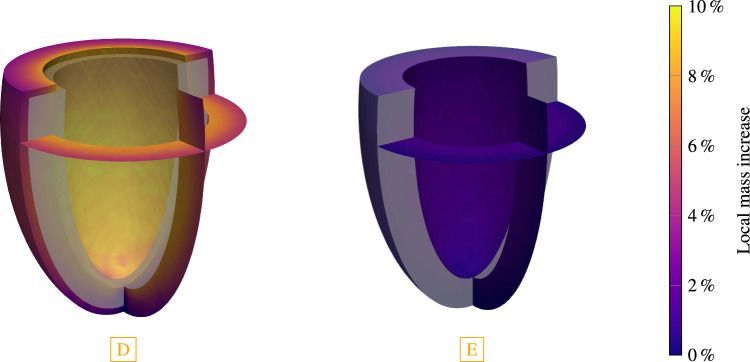
The final configuration of each G&R phase of the simulated stage 1 hypertension. The reference configuration is depicted in gray. Configuration **D** has recovered homeostasis for an increased pressure of $p=140\,{\textrm{mmHg}}$ (stage 1 hypertension). The cavity volume of the ventricle increased and a slightly more spherical shape is observed. Configuration **E** has recovered homeostasis after returning to baseline pressure $p=120\,{\textrm{mmHg}}$. An almost complete reversal of the growth is observed resulting in only a small deviation from the reference configuration

**Fig. 10:**
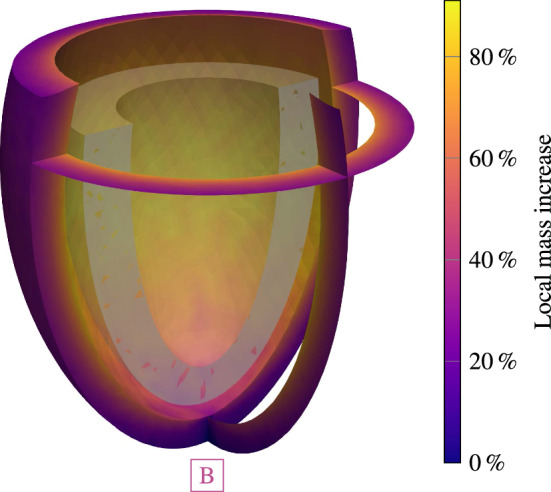
Configuration **B** after stopping unstable G&R for a systolic pressure of $p=180\,{\textrm{mmHg}}$ (stage 2 hypertension). The cavity size increased and the shape renders spherically

In the following, we examine mechanobiological stability of the model of cardiac G&R described in Sect. 2 depending on external loading and mechanobiological key parameters.

To this end, we performed a series of G&R simulations all starting from a prestressed configuration at $p=120\,{\textrm{mmHg}}$ obtained with the procedure described in Sect. 2.5 (using a prestress convergence threshold of $\varepsilon _\text {pre}=0.01\,{\textrm{mm}}$ with a subsequent activation of G&R equations for 100 timesteps to bring the constituents close to homeostasis). Starting from such a configuration, we increased the systolic pressure by a Heaviside step function to a new value $p^+$. We examined the response of the LV to this perturbation in a parameter study. To this end, we varied the growth gain parameters of all remodeling constituents and the systolic blood pressure. The blood pressure was varied between $130\,{\textrm{mmHg}}$ and $240\,{\textrm{mmHg}}$ such that stage 1 and stage 2 hypertension are covered (Chobanian et al. 2003). In the first step, the parameter region of interest, that is, the two-dimensional parameter space $\{(k^i,p^+):~ {0.05}/{T^i} \le k^i \le {0.2}/{T^i};~130\,{\textrm{mmHg}} \le p^+ \le 240\,{\textrm{mmHg}}\}$, is sampled with a spacing of $\varDelta k^i = {0.025}/{T^i}$, $\varDelta p^+ = 10\,{\textrm{mmHg}}$ using a coarse mesh with 2118 quadratic tetrahedral elements. In this first step, it is observed that in one part of this parameter space one observed mechanobiologically unstable G&R, in the other one, however, mechanobiologically stable G&R. To determine the subregion between stability and instability more exactly, the parameter region around the border between stable and unstable G&R is resampled in a second step with a finer spacing in the parameter space. In a last step, the refined border is verified with simulations using the fine mesh with 3970 elements identified in the mesh convergence study.

A simulation is *mechanobiologically stable* if the systems returns to a (new) mechanobiological equilibrium (Cyron and Humphrey 2014) after the perturbation of the external loading. In mechanobiological equilibrium, all constituents are in their preferred stress state, i.e.,

$$\begin{aligned} \lim _{s \rightarrow {} \infty } \frac{|\sigma ^i-\sigma _\text {h}^i|}{\sigma _\text {h}^i} = 0 \quad \forall \quad i. \end{aligned}$$

Otherwise, a simulation exhibits unstable runaway growth and will not converge to a stable homeostatic configuration. We approximate this classification in the context of a standard forward simulation. A simulation is stopped, once the criterion

$$\begin{aligned} \max \frac{|\sigma ^i-\sigma _\text {h}^i|}{\sigma _\text {h}^i} \le \epsilon _{\sigma ,\text {h}} \quad \forall \quad i \end{aligned}$$

is fulfilled (*mechanobiological stability*). If the deviation from homeostasis increases for all constituents, we classify the simulation as *mechanobiologically unstable*:

$$\begin{aligned} \frac{\textrm{d}}{\textrm{d}s}\frac{|\sigma ^i-\sigma _\text {h}^i|}{\sigma _\text {h}^i} > 0 \quad \forall \quad i. \end{aligned}$$

We ran all samples long beyond the point of the stability or instability stopping criteria as given above. None of the samples changed its classification back to instability once the instability criterion was fulfilled or vice versa within the considered timeframe.

Figure 7 visualizes the classification of each sample. In case of a slightly elevated pressure ($p^+ \le 135\,{\textrm{mmHg}}$ ) G&R is stable for all studied growth constants. In contrast, a highly elevated pressure ($p^+ \ge 225\,{\textrm{mmHg}}$) always results in unstable growth. In between, stability depends on the growth constant, i. e., how fast the heart can adapt to the change in LV systolic pressure. The higher the growth constant $k^i \cdot T^i$, the higher the range of increased pressure that yields a stable configuration.

We have shown the influence of the systolic pressure and the growth constant on mechanobiological stability. In the supplementary materials, we show that the stiffness of the spring boundary conditions also influences mechanobiological stability. These results show that the higher the spring stiffness, the smaller is the deviation of the fiber Cauchy stress from homeostasis. The stiffer boundary conditions can bear more of the load. As a consequence, the results of G&R are less pronounced (smaller endocardial diameter and thinner mid-cavity wall thickness). The mechanobiological stability region is larger if the spring stiffness is higher.

In Fig. 8, we focus on one sample of each region, stable and unstable. For both cases, we assume a growth constant of $k^\text {i} \cdot T^i=0.1$, i. e., the same material properties. Both cases differ just by the applied LV systolic pressure increase starting from a baseline homeostatic pressure of $p=120\,{\textrm{mmHg}}$. For the stable case, we assume a systolic blood pressure of $p^+=140\,{\textrm{mmHg}}$ which would be classified as a stage 1 hypertension (Chobanian et al. 2003). For the unstable case, we assume an increase of $50\,{\%}$ ($p^+=180\,{\textrm{mmHg}}$) which would be classified as stage 2 hypertension.

Figure 8 shows for both cases the applied systolic pressure, the mean of the Cauchy fiber stress of cardiomyocytes at all Gauss points with standard deviation, the relative change of the wall thickness in the mid-cavity, the endocardial diameter over time, and the collagen mass fraction. In this section, we discuss stage 1 hypertension (yellow curve) and stage 2 hypertension (purple curve) in the time interval of the increased pressure ($s \in [0, 1325]\,{\textrm{days}}$).

The sudden increase in left ventricular systolic blood pressure results in an elastic elongation of the fibers and therefore in increased stress. In case of stage 1 hypertension, the cardiomyocyte fiber stress increases from the homeostatic value of $22\,{\textrm{kPa}}$ to $22.9 \pm 0.3\,{\textrm{kPa}}$ (mean ± standard deviation). The discontinuity in the wall thickness and the endocardial diameter stems from the elastic elongation of the ventricle due to the sudden pressure increase. The cardiomyocytes and, respectively, the collagen fibers (shown in supplementary material) are not in their homeostatic state after the change in external loading and the G&R evolution equations stimulate G&R of all fiber constituents. The stress of cardiomyocytes and collagen fibers decay exponentially toward their homeostatic stress resulting in a stable limit configuration. In this configuration, the mid-cavity wall thickness decreased to $9.5\,{\textrm{mm}}$ ($-12\,\%$ compared to the reference configuration). The endocardial diameter increased to a value of $45\,{\textrm{mm}}$ ($+20\,{\%}$). Collagen fiber mass fraction in the myocardium also increased from initially $10\,\%$ to $12 \pm 1\,\%$ (mean ± standard deviation). In supplementary material, we also show the evolution of the mass fractions of cardiomyocytes and all collagen fiber families individually. The deposition of collagen fibers is inhomogeneous within the myocardium (not shown) where the maximum is at the endocardium and the minimum at the epicardium. Figure 9a depicts the configuration with the local mass change encoded in color. The reference configuration is also drawn in light gray. Mass is mainly produced in the endocardium, where mass increased by about $10\,{\%}$.

The elastic deformation due to stage 2 hypertension results in a sudden increase in the Cauchy stress of cardiomyocytes to $25.0 \pm 1.2\,{\textrm{kPa}}$. The elastic change of the mid-cavity wall thickness and the endocardial diameter is larger than in stage 1 hypertension. First, the activation of the G&R equations lets the Cauchy stress decay toward the homeostatic stress. However, Cauchy stress plateaus above the homeostatic stress with a subsequent increase. The simulation is stopped when the deviation from homeostasis increases for all constituents. At this point, the mid cavity wall thickness continuously decreased to $7.4\,{\textrm{mm}}$ ($-30\,\%$ compared to the reference wall thickness) and the endocardial diameter increased to $68\,{\textrm{mm}}$ ($+79\,{\%}$). Collagen mass is also excessively deposited resulting in a mass fraction of $25 \pm 5\,\%$ in the final configuration. The final (unstable) configuration is shown in Fig. 10. Locally at the endocardium, the mass has increased by about $92\,{\%}$ compared to the reference configuration. The mass increase is lower at the epicardium.

### Reversal

In the following section, we study to what extent G&R can be reversed when the pressure returns to its baseline value. Physiologically, this corresponds to an intervention that treats hypertension by lowering blood pressure, e g., through ACE inhibitors.

In this section, we focus on the intervals with pressures restored to baseline values in Fig. 8 (i.e., for stage 1 hypertension the interval $s > 1325\,{\textrm{days}}$, i.e., after [D], and for stage 2 hypertension (with early intervention) the interval $s > 150\,{\textrm{days}}$, i.e., after [A]). The starting point for the case stage 1 hypertension is a stable grown configuration as discussed in Sect. 3.2 (yellow, configuration [D]). The elastic response of returning systolic pressure to the physiological level is an immediately decreased cardiomyocyte fiber stress ($21.0 \pm 0.3\,{\textrm{kPa}}$), a slight increase in wall thickness and a slight decrease in endocardial diameter. The activation of the G&R lets the cardiomyocyte fiber stress return exponentially to its homeostatic value. The wall thickness of the mid cavity increases slightly above the reference wall thickness ($+2.0\,{\%}$) and to an almost identical endocardial diameter ($-1.6\,{\%}$) as in the reference configuration. Collagen mass fraction also returned to the baseline value of $10\,\%$. The final configuration [E] is depicted in Fig. 9. It shows only small differences to the reference configuration depicted in light gray.

**Fig. 11:**
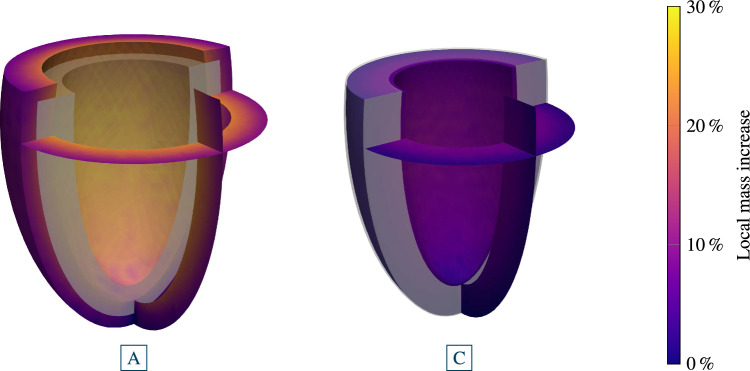
Stage 2 hypertension with an early intervention after $150\,{\textrm{days}}$. The ventricle was loaded with a systolic pressure of $180\,{\textrm{mmHg}}$. Configuration **A** after $150\,{\textrm{days}}$ of G&R. The shown configuration is not in a homeostatic state. Configuration **C** after returning pressure to baseline $p=120\,{\textrm{mmHg}}$ and reaching a homeostatic state. Irreversible changes occurred, resulting in an increased wall thickness

As shown in Sect. 3.2, stage 2 hypertension resulted in unstable growth. Here, we simulated the reversal of G&R that occurs after an early intervention, more precisely a decrease of the systolic pressure to the baseline value after $150\,{\textrm{days}}$ of continuous G&R. We focus on the blue curve in the time interval $s > 150\,{\textrm{days}}$. The elastic response of the pressure normalization results in a sudden decrease of the cardiomyocytes stress to $20.5 \pm 0.5\,{\textrm{kPa}}$, a step increase to a wall thickness of $9.6\,{\textrm{mm}}$ and a slight decrease of the endocardial diameter. In the subsequent G&R phase, the cardiomyocytes stress exponentially increased toward the homeostatic stress. The mid-cavity wall thickness stabilized at $11.8\,{\textrm{mm}}$ ($+10\,{\%}$ compared to the reference configuration). Compared to the baseline collagen mass fraction ($10\,\%$), a slightly increased collagen mass fraction is observed $11 \pm 1\,\%$ in the final state. The endocardial diameter of the final (stable) configuration is $35\,{\textrm{mm}}$ ($-8\,{\%}$). The final configuration [C] is depicted in Fig. 11, with the reference configuration in light gray.

## Discussion

In this work, we developed a computational model of organ-scale cardiac G&R that is based on mechanobiological principles and motivated by myocardial microstructure. We modeled G&R as a consequence of continuous deposition and degradation of sarcomeres and collagen using the homogenized constrained mixture theory and applied it on an idealized 3D left ventricular geometry. We then analyzed mechanobiological stability of our G&R model depending on key mechanobiological parameters and external loading. Additionally, we demonstrated that our model captures the partial reversal of G&R if systolic pressure is returned to its baseline value.

### Prestress

To start our simulations from a state that is in mechanical and homeostatic equilibrium, we introduced a prestressing algorithm which puts the myocardium in a mechanobiologically equilibrated state for a given ventricular pressure. We demonstrated that our prestressing algorithm yields a physiologically meaningful distribution of elastin prestretch.

We assumed in this exploratory study that on the macroscopic scale, the preferred mechanical environment of the constituents is defined by mechanical stress. We critically note, however, that the question which quantity is the target of homeostasis remains unclear and part of ongoing research (Eichinger et al. 2021b). Another heuristic assumption in our study was that systolic stress defines the target state, while it also remains poorly understood how the homeostatic state should be defined and understood best in highly dynamic systems such as the heart.

### Mechanobiological stability

While cardiac G&R can be a physiological response to exercise or pregnancy, pathological G&R plays a crucial role in a variety of cardiac diseases. Adaptive short-term G&R can stabilize cardiac performance to yield a new compensated equilibrium. Yet, in many patients, adverse long-term G&R is mechanobiologically unstable and progresses to heart failure. Identifying the hallmarks of heart failure to select the best therapy is an urgent clinical challenge (Kim et al. 2018). Unfortunately, heart failure lacks robust clinical predictors because the links between biomechanical stimuli and adverse G&R are still unclear (Opie et al. 2006). There is a pressing need to identify the stimuli of adverse G&R and predict the propensity of patients with myocardial infarction to develop heart failure. Computational models of full-heart cardiac G&R, informed by cardiac magnetic resonance imaging, show high potential to fill this gap and to uncover links between biomechanics and cellular mechanisms in a controlled virtual environment (Niestrawska et al. 2020).

Previous models of cardiac G&R based on the kinematic growth theory a priori prescribed the direction and extent of growth. Witzenburg and Holmes (2017) tested six different cardiac G&R models based on the kinematic growth theory and found that none was able to return to homeostasis after mixed pressure and volume overload conditions. In our model, the direction and extent of G&R emerge naturally from intra- and extracellular turnover processes in myocardial tissue constituents. Interpreting the increase in left ventricular pressure as a perturbation of the myocardium’s initial healthy homeostatic state, we demonstrated that our model can predict both mechanobiologically stable and unstable cardiac configurations. Importantly, these configurations can either arise from changes in key parameters (like the product of the mass production parameter $k^i$ and the turnover time $T^i$) or even for an identical model parameter set due to different levels of hypertension. The latter observation is particularly interesting because it extends studies of mechanobiological stability beyond the small perturbation regime studied in Cyron and Humphrey (2014). If the pressure perturbation in our system is large enough, the system is pushed out of the stable regime and subsequent G&R does not converge toward the original or also a new stable mechanobiological equilibrium state but rather continues in an unbounded manner. We can thus identify parameter regions for a patient’s heart where it is more likely to return to a stable equilibrium through G&R. The observation that fast adaption rates can stabilize G&R was previously made for arterial tissue as well (Latorre and Humphrey 2019).

### Reversal

Living tissue such as the myocardium can increase and decrease in size. However, many existing computational models have focused their modeling on mass increase. If only the pathological case is of interest, this would be sufficient. However, there are medications and medical devices that aim at stopping or even reversing cardiac G&R. In clinical practice, it is, thus, of interest if and how well a patient responds to a certain therapy, which necessitates that reverse remodeling is also captured by a model. We demonstrated that our model can inherently describe also the reversal of G&R.

We have analyzed the reversal of G&R in two scenarios in detail. The reversal of a stabilized stage 1 hypertrophy resulted almost in the original configuration. This means that during the preceding hypertension period, mainly reversible G&R occurred. In the case of unstable stage 2 hypertension case, we simulated an early intervention after $150\,{\textrm{days}}$ following the increased pressure. Here, the final reversed configuration is distinct from the reversal of stage 1 hypertension: An increased wall thickness remains with a decreased cavity size and a significant amount of fibrosis. The G&R that occurred in the preceding unstable hypertension period partially resulted in irreversible G&R, despite the pressure returning to its baseline value. This is also observed in real hearts: Adaptive growth due to high blood demand (e.g., during exercise or pregnancy) is also termed *reversible growth*, whereas maladaptive growth is termed *irreversible growth*.

### Phenotype

In our simulations, hypertension in the left ventricle resulted in dilation of the ventricle with a reduction in wall thickness — typically termed eccentric hypertrophy. Left ventricular G&R following a pressure overload is often experimentally analyzed with aortic banding experiments (Imamura et al. 1990; Roussel et al. 2008). These experiments typically show an increase in wall thickness a few days to weeks after banding, hence, mainly concentric hypertrophy. In clinical practice, this pattern is less obvious. Typically, a mix of both patterns, concentric and eccentric hypertrophy can be observed. Cuspidi et al. (2012) found evidence that eccentric hypertrophy was more prevalent than concentric hypertrophy in their examined studies. It is important to note that co-pathologies often influence the observations in clinical practice.

Currently, our model only considers the end-systolic configuration of the heart cycle. Growth stimuli are, however, active in the whole cardiac cycle. Our model currently cannot detect the difference between growth stimuli of pressure and volume overload and, therefore, cannot yet represent both concentric and eccentric hypertrophy.

Constrained mixture type models consider G&R of different constituents individually. Our model predicts a significant increase in collagen mass fraction which is also observed in models of rat left ventricles after 4 weeks of aortic banding (Doering et al. 1988). Our model also predicts a gradient in left ventricle fibrosis from endo- to epicardium as observed in patients with systemic hypertension (Cowling et al. 2019). In the severe hypertensive case, our model predicts only a partial reversal of fibrosis which is in line with studies on patients after treatment of advanced hypertension (Frangogiannis 2020).

### Experimental validation

Experimental data for validating microstructural features of our model remain lacking (Humphrey 2021); hence, we rely on the qualitative comparisons in Sect. 4.4. For a more in-depth quantitative validation, experimental data on the microstructural and the organ scale are necessary.

Cyron et al. (2016) have shown that homogenized constrained mixture models can capture results from experiments with tissue equivalents. The results of these experiments, however, strongly depend on the composition of the tissue equivalents making it difficult to deduce material parameters from these experiments for our organ-scale model. Fischer et al. (2019) demonstrated how living myocardium excised from explanted failing hearts can be analyzed in a biomimetic environment. Analyzing long-term G&R with such an experimental setup might one day allow for calibrating our G&R model with species-specific parameters of the heart.

To validate the model on the organ scale, longitudinal imaging data of cardiac G&R is necessary. Stoeck et al. (2021) analyzed the response of the myocardium to infarction on a porcine model. Their results could be used to verify a model of post-myocardial infarction G&R. We cannot validate our model against their results since modeling of myocardial infarction has not yet been done, but it is one of our following projects.

### Computational performance

The computational cost of homogenized constrained mixture models is comparable to the one of kinematic growth models and much lower than typically for classical constrained mixture models based on multi-network theory (Humphrey 2002). Latorre and Humphrey (2018) recently proposed an interesting approach to speed up computations based on classical constrained mixture models that directly yields the long-term outcome of G&R. However, with this kind of model, reversal of an intermediate configuration (early intervention) is not possible making homogenized constrained mixture models the best available trade-off between computational cost and physiological realism for our study.

The computational costs of the prestressing algorithm are comparable to the one of the successive G&R simulation. However, prestressing has to be done only once per geometry and the resulting configuration can be used to simulate multiple G&R scenarios.

With our, so far, not optimized implementation, we ran up to 8 different G&R scenarios on one Intel Xeon E5-2630 v3 “Haswell” (16 cores, $2.5\,{\textrm{GHz}}$, $64\,{\textrm{GB}}$) of our Linux cluster. The prestress algorithm needed around $33\,{\textrm{min}}$ ($\varepsilon _\text {pre}=0.01\,{\textrm{mm}}$) to obtain a mechanobiological equilibrated reference configuration. The prestress algorithm had on average 3.2 Newton iterations per step and 219 linear solver iterations per Newton iteration. The subsequent G&R took around $1\,{\textrm{h}}$ for the first $250\,{\textrm{days}}$ of G&R. Stage 1 hypertension had on average 3.4 Newton iterations per time step and 273 linear solver iterations per Newton iteration with a downward tendency while approaching homeostasis. For Stage 2 hypertension, these values were 3.9 and 389, respectively, with a tendency to rise during runaway growth.

### Limitations and future perspectives

Our simulations were conducted on a truncated spheroid as an approximation of the left ventricle of the heart. The geometry of the heart also influences G&R in the ventricles. In general, homogenized constrained mixture models are not limited to regular geometries. Mousavi et al. (2019) analyzed the evolution of ascending thoracic aortic aneurysms on patient-specific geometries using the homogenized constrained mixture model. A computational model of cardiac G&R that bases on the homogenized constrained mixture model on patient-specific geometries is one of our next steps toward the goal of clinical applications.

We only differentiated between mechanobiologically stable and unstable G&R. However, there are cases where the shape change is too deleterious, and heart failure occurs despite G&R reaching a stable limit, or in contrast, cases where unstable G&R occurs on a very long time scale so that the deleterious shape change would occur long after the natural death of the patient. A multi-timescale simulation with the G&R scale and the heart cycle scale is necessary to capture the transient effects of heart failure. Here, the simulation of several cardiac contractions could be sped up with reduced-order models (Pfaller et al. 2020).

We describe myocardial tissue as a constrained mixture of structurally relevant constituents, each having a different prestretch. Material parameters for this class of models for the myocardium are lacking. Standard material models account only for the overall stress response without incorporating different constituents and their prestretch. Ex vivo experimental data on myocardial tissue (Sommer et al. 2015) can be used to identify a set of parameters of a prestressed constrained mixture.

Currently, we use the same model for turnover of cardiomyocytes and collagen fibers. Constrained mixture type models can, however, use individual models for different constituents. Since turnover in cardiomyocytes is an intracellular process (Willis et al. 2009), the microstructural processes differ from the observations of collagen turnover. Individual models for each constituent could be developed which are based on constituent-specific assumptions and microstructural observations.

There are currently no reliable models for entropy influx during G&R of soft tissue. As a consequence, we cannot discuss the thermodynamic consistency of the model in a strong sense. Constraints imposed by the Clausius–Duhem inequality (second law of thermodynamics) need an increased theoretical understanding of soft tissue G&R (Humphrey 2021).

Currently, we assumed that only the myocardial tissue itself adapts to changed loads. We assumed that the behavior of the pericardial boundary condition, the cut-off of the atria and the hemodynamic loads do not change over time. However, the pericardium is known to enlarge in size and shape during cardiac dilatation (Freeman and LeWinter 1984) and the atria will also adapt to changes in the mechanical environment. To model the adaption of the atria and the right ventricle, a four-chamber geometry of the heart could be used. Modeling the change in hemodynamics is, however, more involved. Models have been developed to describe the regulation of blood pressure on the long timescale (Beard et al. 2013). Recently, Pourmodheji et al. (2022) coupled right ventricular and pulmonary arterial G&R in a multi-temporal computational model.

As discussed in Sect. 4.4, cardiac G&R concerns two time-scales, G&R (days) vs. a single heartbeat (seconds). In our work thus far, we have only considered the long-term G&R scale. However, biomechanical loads on myocardial constituents change significantly over one cardiac cycle. In future work, we plan to extract G&R stimuli from the transient load through the cardiac cycle. This will allow our model to better distinguish between the G&R patterns of concentric and eccentric hypertrophy.

A global sensitivity analysis (Brandstaeter et al. 2021) of our model could identify parameters with a high influence on mechanobiological stability. This could provide answers for the clinically relevant question of whether a patient with specific features (shape of the heart, composition of the myocardium, overload conditions, etc.) is at risk of unstable G&R.

### Supplementary Information

Below is the link to the electronic supplementary material.

Supplementary file 1 (pdf 293 KB)

## Data Availability

All data generated or analyzed during this study are included in this published article.

## Footnotes

1 We use *s* for the G&R time to emphasize the difference to the timescale of a heartbeat usually denoted as *t*.

2 Note that no fibers are under compression in the examples within the paper.
