# Supplementary material for: A homogenized constrained mixture model of cardiac growth and remodeling: analyzing mechanobiological stability and reversal
Source: Biomech Model Mechanobiol. 2023 Jul 23;22(6):1983–2002. doi: 10.1007/s10237-023-01747-w (PMC10613155; doi:10.1007/s10237-023-01747-w)
Supplement: Supplementary file 1 — (pdf 293 KB) [file 10237_2023_1747_MOESM1_ESM.pdf]

Supplementary material for: A homogenized constrained mixture model of cardiac growth and remodeling: Analyzing mechanobiological stability and reversal

1 Details of tissue turnover by constituent

Figure 1 shows the mass fraction of each G&R fiber (myocytes and 4 collagen fiber families) for the simulation cases shown in Figure 8 of the manuscript. The average mass fraction of each collagen fiber family within the myocardium increase similarly. Collagen deposition mainly happens near the endocardium. The standard deviation is smaller for collagen fiber family 3 since this family is lower since the family is approximately oriented in the longitudinal direction of the heart at the endocardium where most of the collagen is deposited. The singularity of the fiber direction is, therefore, less pronounced resulting in smaller deviations within the myocardium.

Although the mass fraction of cardiomyocytes decreases, the mass itself increases within the myocardium resulting in an enlargement of individual cardiomyocytes. Figure 2 shows the fiber stress of each collagen fiber family within the myocardium. Their convergence behavior is similar to the Cauchy stress of cardiomyocytes reported in the manuscript. As for the collagen mass fraction, the difference in the standard deviation between the collagen fiber families stems from the less pronounced effect of the singularity of the fiber orientation at the endocardial apex for collagen fiber family 3.

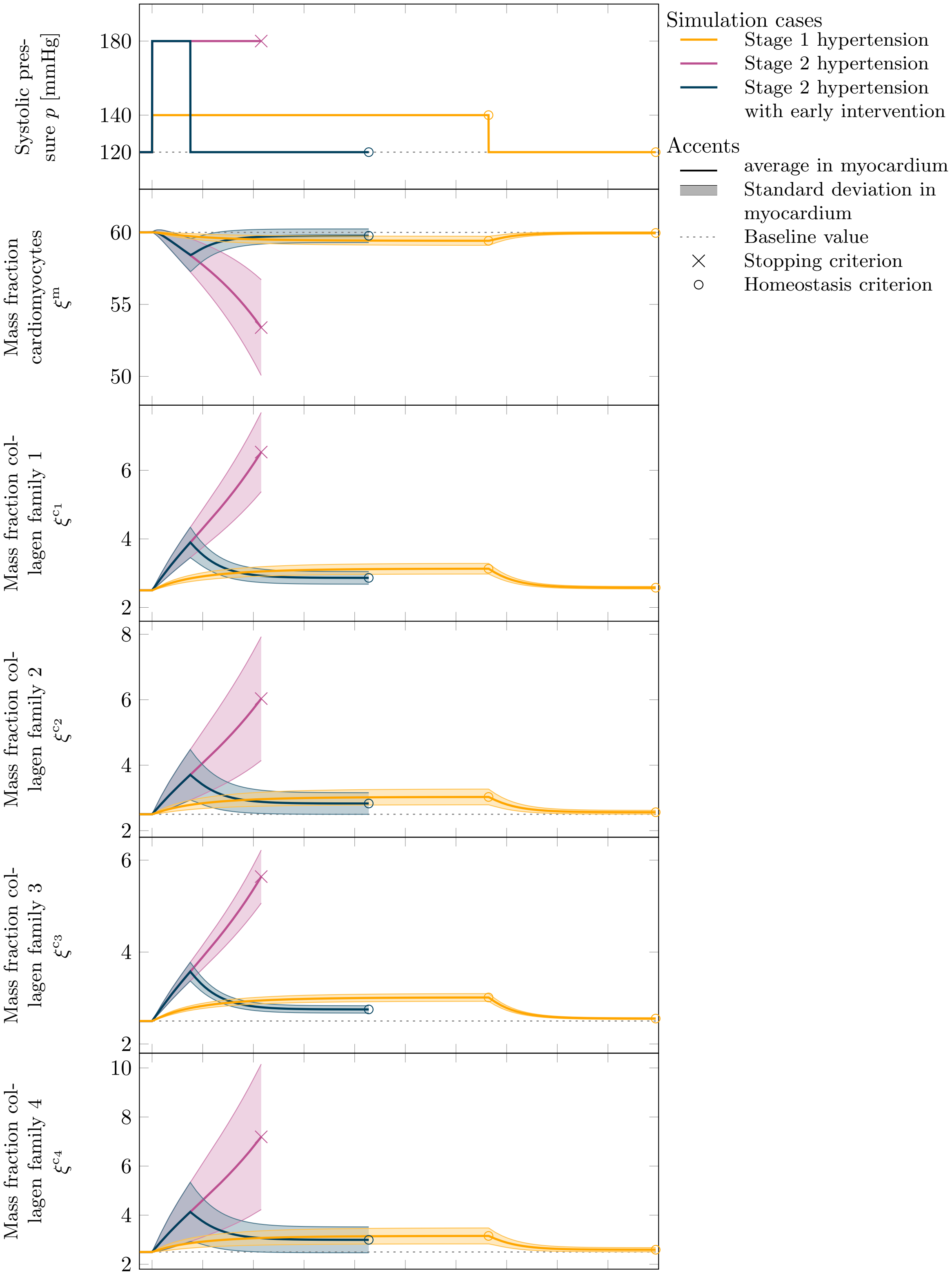

Figure 1: Evolution of the mass fraction of each fiber constituent (cardiomyocytes and collagen fiber families) with the standard deviation within the myocardium for the simulation cases shown in Figure 8 of the manuscript.

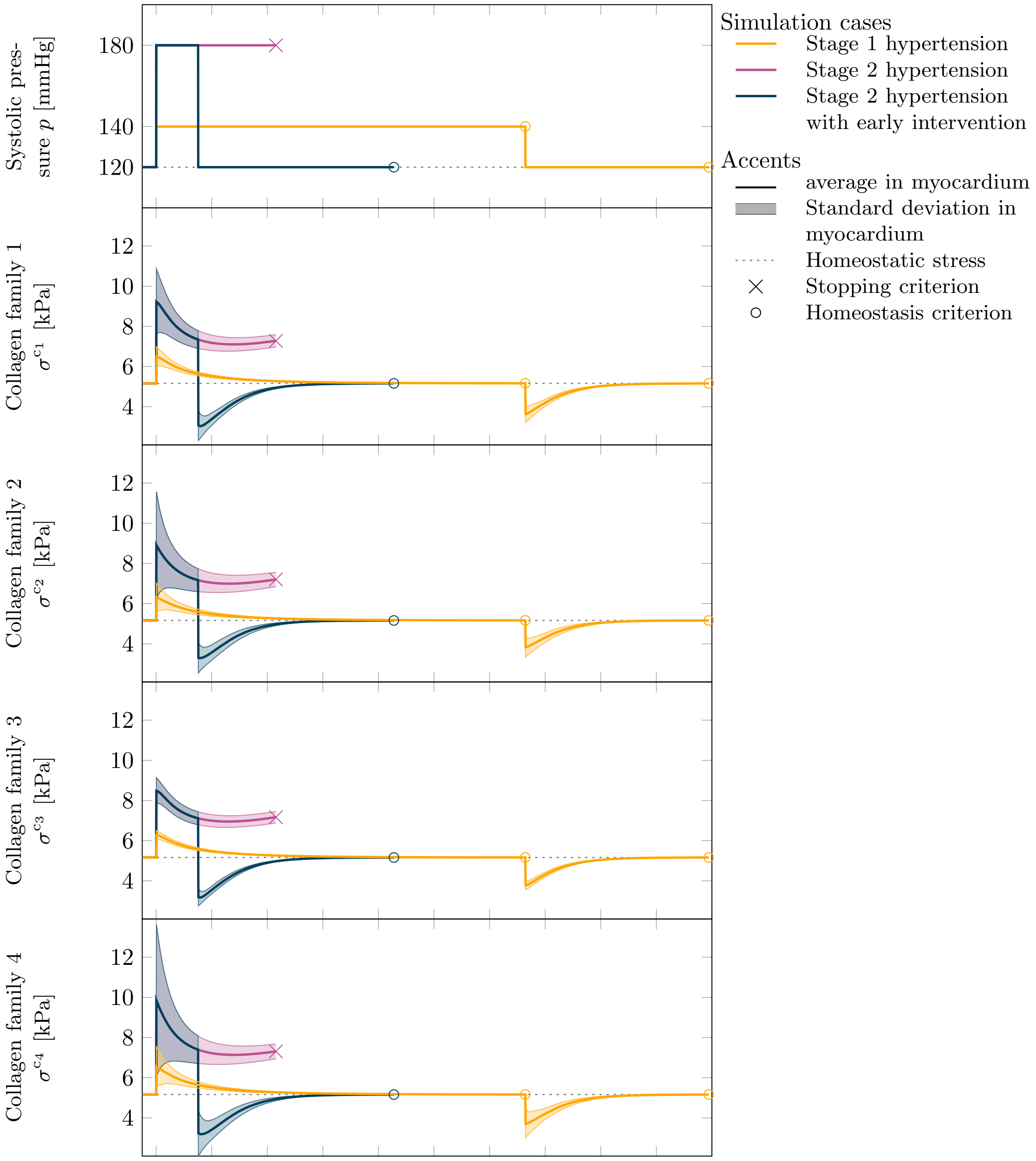

Figure 2: Evolution of the Cauchy stress of all collagen fiber families with the standard deviation within the myocardium for the simulation cases shown in Figure 8 of the manuscript.

2 Influence of spring stiffness

The Figures 3 and 4 show the influence of the spring-stiffness on G&R. The stiffness of the springs at the base and at the epicardium are scaled by κ with $k^i T^i = 0.1$. Figure 3 and Figure 4 depict stage 1 and stage 2 hypertension, respectively. The deviation from homeostasis gets smaller for stiffer spring boundary conditions. As a result, G&R is less pronounced (smaller endocardial diameter and thinner mid-cavity wall thickness). This also influences mechanobiological stability. For $k = 0.33$, even (previously stable) stage 1 hypertension results in unstable G&R as the pericardium provides too little support for stabilization. In contrast, (previously unstable) stage 2 hypertension can result in stable G&R for $\kappa \geq 9$.

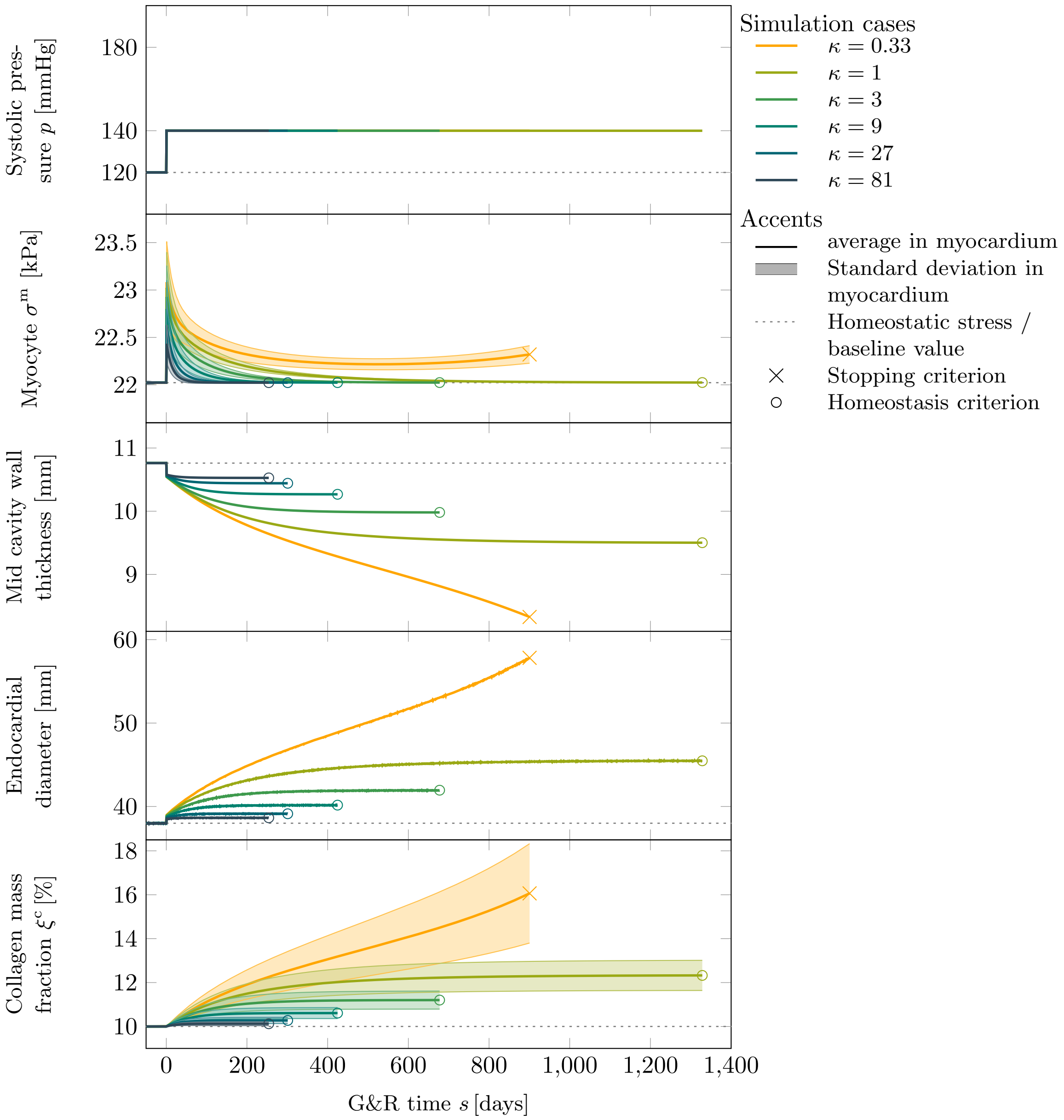

Figure 3: Simulation of stage 1 hypertension with scaled spring stiffness for the boundary condition at the pericardium and the base. Both stiffnesses are scaled by κ. Depicted is the systolic pressure, the myocyte stress with the standard deviation in the myocardium, the mid-cavity wall thickness, the endocardial diameter and the collagen mass fraction. For $\kappa = 0.33$, the support by the pericardial boundary conditions is too small such that G&R is unstable even for stage 1 hypertension .

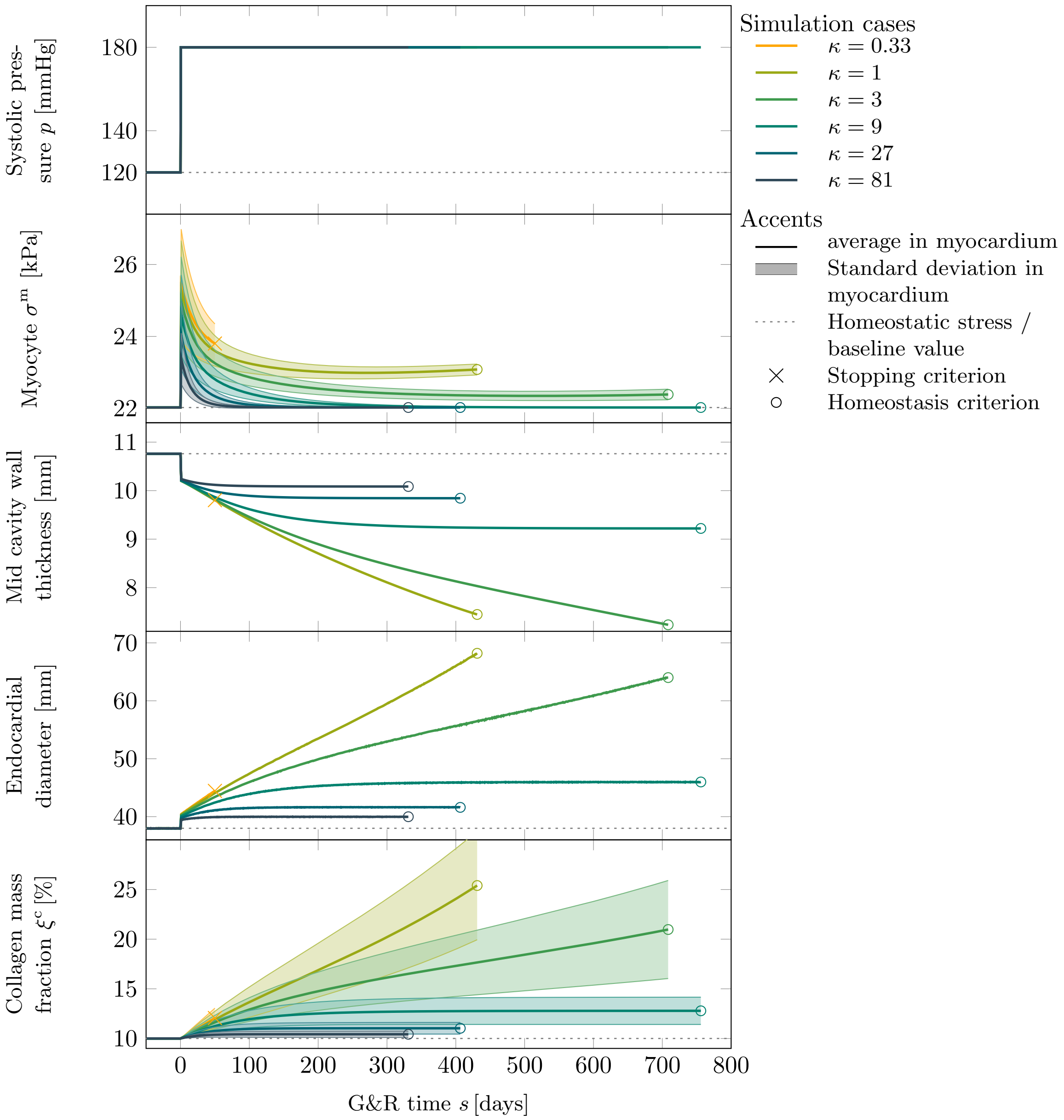

Figure 4: Simulation of stage 2 hypertension with scaled spring stiffness for the boundary condition at the pericardium and the base. Both stiffnesses are scaled by κ. Depicted is the systolic pressure, the myocyte stress with the standard deviation in the myocardium, the mid-cavity wall thickness, the endocardial diameter and the collagen mass fraction. For a large enough stiffness ($\kappa \geq 9$), stage 2 hypertension can also result in stable G&R.
